# Patient-centered feedback on the results of personality testing increases early engagement in residential substance use disorder treatment: a pilot randomized controlled trial

**DOI:** 10.1186/s13722-015-0030-9

**Published:** 2015-03-14

**Authors:** Daniel M Blonigen, Christine Timko, Theodore Jacob, Rudolf H Moos

**Affiliations:** Center for Innovation to Implementation, Veterans Affairs Palo Alto Health Care System, 795 Willow Road (152-MPD), Menlo Park, CA 94025 USA; Family Research Center, VA Palo Alto Health Care System, 795 Willow Road (152-MPD), Menlo Park, CA 94025 USA; Palo Alto University, 1791 Arastradero Road, Palo Alto, CA 94304 USA; Department of Psychiatry & Behavioral Sciences, Stanford University School of Medicine, 401 Quarry Road, Palo Alto, CA 94304 USA

**Keywords:** Substance use disorders, Patient-centered care, Therapeutic assessment, Treatment engagement, Personality testing, Feedback

## Abstract

**Background:**

Patient-centered models of assessment have shown considerable promise for increasing patients’ readiness for mental health treatment in general, but have not been used to facilitate patients’ engagement in substance use disorder (SUD) treatment. We developed a brief patient-centered intervention using assessment and feedback of personality data and examined its acceptability and efficacy to increase early engagement in residential SUD treatment.

**Methods:**

Thirty patients entering a 90-day residential SUD treatment program were randomly assigned to a feedback (n = 17) or control (n = 13; assessment-only) condition. Normal-range personality was assessed with the NEO Personality Inventory-Revised (NEO PI-R). Patients were re-interviewed one month after treatment entry to obtain information on their satisfaction with the intervention, as well as their adjustment to the residential milieu. Electronic medical records were reviewed to obtain information on patients’ length of stay in the program and discharge status. Univariate ANOVAs and chi-square tests were conducted to examine group differences on outcomes.

**Results:**

Patients’ ratings indicated strong satisfaction with the feedback intervention and expectations that it would have a positive impact on their treatment experiences. Among patients who had not previously been treated in the residential program, the feedback intervention was associated with more positive relationships with other residents in treatment and a stronger alliance with the treatment program one month after treatment entry. The feedback intervention was also associated with a longer length of stay in treatment, although this effect did not reach statistical significance.

**Conclusions:**

The findings highlight the clinical utility of providing SUD patients with patient-centered feedback based on the results of personality testing, and provide preliminary support for the acceptability and efficacy of this intervention to facilitate early engagement in residential SUD treatment.

## Background

Engagement in substance use disorder (SUD) treatment is a robust predictor of treatment retention and outcomes [1,2]. Measures of positive adjustment, such as more patient satisfaction [3], perceived program support and alliance [4,5], and supportive relationships with peers in treatment [6], may be conceptualized as indicators of treatment engagement [7] and are linked to better outcomes and a longer length of stay in SUD treatment.

Personality traits have also been linked to SUD treatment engagement and retention [8,9]. However, the field has lacked a compelling model for how to use information about personality to facilitate patients’ engagement in treatment. Patient-centered models of assessment and feedback, such as Finn’s semi-structured approach (“therapeutic assessment”) [10,11], are well suited for this purpose. In this approach, patients are viewed as collaborators who work with an assessor to define assessment goals and help interpret assessment results. Use of assessment results in this manner is theorized to facilitate positive change in patients by addressing needs of self-verification, self-enhancement, and self-discovery [12]. The efficacy of this approach for increasing readiness for and engagement in mental health treatment is supported by quasi-experimental and pragmatic trials [13,14], randomized trials [15-17], and a meta-analysis [18]. However, this approach has not been used to facilitate patients’ engagement in SUD treatment.

We developed a patient-centered intervention using assessment and feedback of personality data for patients entering residential SUD treatment. Briefly, upon entry to a residential SUD program, patients are assessed with the NEO Personality Inventory-Revised (NEO PI-R) [19], a measure of normal-range personality, and are provided with personalized feedback describing: (a) how their personality profile compares to that of their age-related peers; (b) their possible pattern of adjustment to the treatment milieu; and (c) cognitive-behavioral assignments that can help address problematic behavioral tendencies. This approach is expected to help patients adapt to the demands of the residential setting by enabling them to anticipate how their personal tendencies may impact their treatment experiences. Further, the assessment of normal-range personality dimensions allows patients to feel recognized as unique individuals rather than as psychiatric patients with a pathological condition. Finally, the assessment results (a description of the patient in “normal” personality terms) are also provided to treatment staff to facilitate their ability to empathize with patients and assist with the development of more individualized treatment plans for patients.

This patient-centered intervention follows several principles of collaborative forms of assessment, particularly the principles of therapeutic assessment espoused by Finn [10,11]. For example, patients work with an assessor to develop individualized assessment questions in terms of what they would like to learn about themselves from the personality assessment, identify behavioral tendencies they would like to work on or change, or how to get the most out of the residential program. To facilitate this, patients are presented with brief descriptions of the personality domains that are measured by the NEO PI-R and are asked if any are of particular interest to them and why. During the feedback session, patients are encouraged to assist with interpretation of the assessment results in terms of how well the test’s description of their personality does or does not match their experiences in the residential milieu and in the context of their lives more generally. Consistent with the tenets of therapeutic assessment, the assessor is encouraged to maintain empathic connections with patients during all sessions and to discuss and acknowledge patients’ past assessment experiences and any reservations they may have about the current assessment process.

In addition to its use of therapeutic assessment principles, this intervention incorporates several elements of a personality assessment system for SUD patients that was developed by Moffett, Steinberg, and Rhode [20]. Specifically, the present intervention involves: (a) the assessment of normal, rather than abnormal, personality traits; (b) feedback to patients on their possible adjustment to the treatment milieu, given their personality profile; and (c) treatment recommendations that may help the patient maximize his/her benefit from the program. Consistent with Moffett et al. [20], with patients’ permission, assessment results are also shared with the staff of the residential program. In contrast to Moffett et al. [20], the present intervention does not include a reassessment of the patient’s personality during treatment in order to revise his or her treatment plan. Rather, it includes a follow-up session with the patient one month into treatment to discuss the consistency of the assessment findings with the patient’s treatment experiences and progress toward treatment goals, and the utility of the previously suggested treatment recommendations.

The objectives of the present study were to examine the acceptability of a patient-centered feedback intervention using assessment and feedback of personality data and efficacy of the intervention to increase early engagement in SUD treatment. This investigation was designed as a pilot randomized controlled trial (RCT) with the goal of obtaining preliminary data on the objectives; thus, it corresponds to a Stage I trial in the stage model of behavioral therapies [21].

## Methods

### Participants

Participants included 30 patients entering a 90-day residential SUD treatment program. Patients were mostly male (n = 28); ranged in age from 26 to 64 years (M = 49.07 years, SD = 11.15); had 13 years of education, on average (SD = 2.27); were predominantly Caucasian (n = 18); and were not currently married (n = 28). On average, patients had three prior episodes of residential SUD care (SD = 2.26).

### Design and procedures

Within one week of entry into the residential program, patients were recruited and randomly assigned to either the feedback (n = 17) or control (n = 13) condition. We oversampled for patients in the feedback condition to maximize information on perceptions of the intervention—i.e., prior to recruitment, a list of 30 numbers (20 corresponding to the feedback condition, and 15 corresponding to the control condition) were put into random order in a Microsoft Excel spreadsheet. As patients entered the study, a research assistant added the patient to the randomization spreadsheet, notified the patient of his/her assignment, and scheduled the initial session with the Intervention Coordinator (IC).

The intervention procedures were conducted by a masters-level clinician who served as the IC. The IC was trained in the feedback protocol by the lead author. The training protocol included mock intervention sessions with the lead author and practice intervention sessions with eight SUD patients from the target program. The lead author was trained in the principles of therapeutic assessment by Dr. Finn and other members of the Center for Therapeutic Assessment through multiple in-person courses.

For the randomized trial, patients in the feedback condition completed three sessions with the IC. Sessions were videotaped and reviewed weekly with the lead author to ensure the IC’s fidelity to the protocol:

- *Initial session* (8.3 mean days after treatment entry, SD = 3.0): Patients completed assessments of sociodemographics, treatment history, substance-related functioning, and personality. Next, patients worked with the IC to develop individualized assessment questions geared toward what they wanted to learn about themselves from the personality assessment, behavioral tendencies they wanted to work on or change, or how to get the most out of the residential program. To facilitate this, patients were presented with brief descriptions of the five broad domains of personality that are measured by the NEO PI-R and were asked if any were of particular interest to them and why. Patients were then asked to rate (a) how they thought their scores on the NEO PI-R compared to those of others their age and gender, and (b) their “ideal” personality. Responses to both sets of questions were given on a 5-point scale (1 = much lower than others my age, 5 = much higher than others my age) and were used by the IC to engage patients in a discussion of their current beliefs about their personality. This approach to administration of the NEO PI-R was used to assist the patient and IC in the development of individualized assessment questions (e.g., “How do I compare to others in terms of self-esteem and anger?”), as well as to help the IC understand aspects of the patient’s personality in which he or she might lack insight. This information was then used by the IC to develop a personalized summary sheet of the assessment results. To maximize the patient’s comprehension, feedback was limited to three or four test findings, with priority given to information that: (a) was relevant to the patient’s assessment questions; (b) matched the patient’s current self-perception; and (c) expanded the patient’s knowledge about his or her personality.
- *Patient-centered feedback session* (13.8 mean days after treatment entry; SD = 4.9): The IC provided patients with a summary sheet describing: (a) how their personality profile compared to that of others their age and gender; (b) how their personality might impact their adjustment to the program; and (c) recommendations to help address problematic behavioral tendencies. An example of feedback given to a patient in the feedback condition is shown in the Appendix. For each assessment finding presented by the IC, the patient was encouraged to assist with its interpretation in terms of how well the description of his/her personality did or did not match his/her experiences in the milieu and life more generally, as well as which behavioral tendencies (as measured by the NEO PI-R) he/she wanted to prioritize in treatment. At the end of the session, a research assistant *not* involved in the patient-centered assessment collected information on the patient’s perceptions of the feedback intervention.
- *1-month follow-up session* (32.8 mean days after treatment entry; SD = 4.8): Patients completed assessments regarding their adjustment to the residential program (these data were also collected by a research assistant who was not involved in the patient-centered assessment). Next, the patient and IC reviewed the summary sheet and discussed consistency of the findings with the patient’s treatment experiences, the patient’s progress towards their treatment goals, and usefulness of the previously suggested treatment recommendations.

In addition, the IC provided feedback to program staff regarding patients’ personality profiles and discussed with staff how to incorporate the recommendations into patients’ treatment plans.

Patients in the control condition received only the initial and 1-month follow-up sessions with the IC, which entailed administration of the same assessments given to patients in the feedback condition. All procedures were approved by the local institutional review board.

### Measures

#### Initial session

*Substance use-related functioning.* A 7-item “risk-use” factor from the Brief Addiction Monitor [22] assessed physical and psychological health, drug use and cravings, exposure to risky situations, and interpersonal problems with family and friends in the past 30 days [23]. Scores could range from zero to 28 (M = 13.23, SD = 4.93; α = .68).

*Personality.* The NEO PI-R [19] is a 240-item self-report measure of normal-range personality. Items were rated on a 5-point scale (1 = strongly disagree, 5 = strongly agree), which provided age- and gender-normed T-scores (relative to the general population) on five factors: Neuroticism – tendency to experience negative affective states (M = 67.07, SD = 12.44; α = .93); Extraversion – tendency to be sociable, assertive, and active (M = 44.50, SD = 10.77; α = .87); Openness – intellectual curiosity and willingness to entertain novel ideals and unconventional values (M = 48.57, SD = 10.90; α = .86); Agreeableness – tendency to be altruistic, cooperative, and sympathetic (M = 45.90, SD = 13.45; α = .90); and Conscientiousness – tendency to be planful, organized, and reliable (M = 35.73, SD = 12.43; α = .94). Each factor also provided scores on six facet scales.

There were no significant differences between participants in the feedback and control conditions on any of the demographic or prior treatment variables, on the substance use-related functioning scale, or on any NEO PI-R facet or factor scores.

#### Feedback session

*Perceptions of the feedback intervention.* The Assessment Questionnaire (AQ), a 48-item questionnaire measuring satisfaction with the patient-centered assessment process [15,17], was administered to patients in the feedback condition only. Items were rated on a 5-point scale (1 = strongly disagree, 5 = strongly agree) and yielded scores on four factors: new self-awareness – how much patients felt that they learned something new about themselves (13 items); positive accurate mirroring – how much patients felt validated and understood by the assessment (12 items); positive relationship with the examiner – how much patients experienced a strong alliance with the IC (12 items); and negative feelings about the assessment – how much patients felt hurt, judged, or exposed by the assessment (11 items). A total satisfaction score was computed from the average response to all 48 items.

Patients also provided ratings on a 5-point scale (1 = very negative effect, 5 = very positive effect) regarding the expected impact of the intervention on their: (a) relationship with program staff; (b) likelihood of following staff recommendations; (c) relationship with other residents; (d) likelihood of following recommendations of other residents; (e) willingness to stay in the program; (f) willingness to continue treatment after leaving the program; and (g) motivation to stay sober from alcohol and other drugs.

#### 1-month follow-up session

*Program adjustment.* For all patients, adjustment to the program was assessed by: (a) satisfaction with the program—i.e., scores on an 11-item version of the Client Satisfaction Questionnaire (CSQ) [24] (α = .95); (b) program support—i.e., the sum of 10 true-false items on the Support subscale of the Community Oriented Programs Environment Scale (COPES) [25] (α = .81); and (c) positive relations with other residents—i.e., scores on the 6-item Resident Resources subscale from the Life Stressors and Social Resources Inventory (LISRES) [26] (α = .84). A program alliance composite was constructed based on the average of the *z*-scores of the CSQ, COPES, and LISRES scales, which were highly intercorrelated (average *r* = .55; range = .40–.76) [4].

*Treatment outcomes.* Information regarding length of stay and whether or not patients dropped out of the program was gathered from administrative records 3 months after patients’ dates of entry.

## Results

### Perceptions of the feedback intervention (Table 1)

**Table 1:** **Perceptions of the feedback intervention**

**Variable**	**M**	**SD**	**Range**	**α**
*Satisfaction with the patient-centered assessment (Assessment Questionnaire: T-scores)*				
Factor 1 – New self-awareness	51.53	7.01	34.34–60.91	.95
Factor 2 – Positive accurate mirroring	52.52	4.17	44.44–59.44	.90
Factor 3 – Positive relationship with the examiner	56.01	5.21	43.70–62.22	.84
Factor 4 – Negative feelings about the assessment	50.00	3.57	46.11–58.23	.85
Total satisfaction	54.91	4.08	45.68–62.16	.93
*Perceived impact of feedback intervention (1 = very negative effect, 5 = very positive effect)*				
Relationship with program staff	4.29	0.77	3–5	--
Likelihood of following staff recommendations	4.24	0.83	3–5	--
Relationship with other residents	4.41	0.62	3–5	--
Likelihood of following recommendations of other residents	4.12	0.60	3–5	--
Willingness to stay in the program	4.47	0.94	2–5	--
Willingness to continue treatment after leaving the program	4.53	0.80	3–5	--
Motivation to stay sober from alcohol and other drugs	4.47	0.72	3–5	--

Notes. n = 17.

T-scores for the AQ factors, based on norms for patients treated at the Center for Therapeutic Assessment (Finn, personal communication, 8 Nov 2011), ranged from 50.00 to 56.01, indicating that patients were highly satisfied with the intervention. Mean ratings on items assessing the expected impact of the feedback ranged from 4.12 to 4.53 (scale of 1–5), indicating that patients thought the intervention would have a positive impact on their experiences in the program.

### Program adjustment and treatment outcomes (Table 2)

**Table 2:** **Program adjustment and treatment outcomes across the feedback and control conditions**

	**M (SD) or % (** ***n*** **)**			
**Outcome variable (measure)**	**Feedback**	**Control**	**F or χ** ^**2**^	**Mean ES difference (** ***d*** **)**
*Program adjustment (1-month) (M, SD)*				
Satisfaction with the treatment program (CSQ)	37.29 (6.61)	33.11 (10.34)	1.59	0.48
Program support (COPES)	7.71 (2.59)	6.00 (2.96)	2.32†	0.61
Positive relations with other residents (LISRES)	18.29 (4.77)	14.89 (4.70)	3.03*	0.72
Program alliance composite	0.19 (0.74)	-0.45 (1.00)	3.39*	0.72
*Treatment outcomes*				
Length of stay in treatment (days) – (M, SD)	82.47 (18.84)	75.56 (34.24)	0.51	0.25
Premature dropout from the program – % (*n*)	29.4 (5)	33.3 (3)	0.42	--

Notes. Feedback condition (n = 17), control condition (n = 9). Degrees of freedom: F tests (1, 24), χ^2^ test (1). †p < .10 (one-tailed), *p < .05 (one-tailed). CSQ = Client Satisfaction Questionnaire. COPES = Community Oriented Programs Environment Scale. LISRES = Life Stressors and Social Resources Inventory. The program alliance composite represents the average of the standardized (z) scores on the CSQ, COPES, and LISRES indices.

Univariate ANOVAs and chi-square tests compared the feedback and control conditions on program adjustment (1-month) and treatment outcomes. Four patients (all from the control condition) who had previously attended the program were excluded from these analyses to balance the conditions in terms of having no prior exposure to the treatment milieu. Patients in the feedback condition had higher ratings on all program adjustment indices, with generally large effect sizes (average Cohen’s *d* = .63; range = .48–.72). Patients in the feedback condition reported significantly more positive relations with other residents and had significantly higher scores on the program alliance composite. There were no statistically significant differences between conditions on treatment outcomes. However, those in the feedback condition had a longer length of stay in the program (i.e., approximately 7 days, on average) than those in the control condition, which equated to a small to medium effect size.

## Discussion

This study represents a Stage I efficacy [21] trial of a patient-centered feedback intervention based on the results of personality testing, which aimed to increase patients’ engagement in SUD treatment. This intervention is novel because of its application of therapeutic assessment principles to an SUD population, as well as its integration of these principles with the personality assessment system for SUD patients developed by Moffett and colleagues [20]. The findings provide preliminary support for the acceptability and efficacy of this intervention to facilitate early engagement in SUD treatment and highlight the clinical utility of providing patient-centered feedback on personality testing to SUD patients. Accordingly, this work lays the foundation for larger trials with additional follow-up assessments to test the efficacy of this intervention to increase retention in SUD care and improve outcomes post-treatment (i.e., a Stage II trial) [21].

While encouraging, the findings should be interpreted with caution, given the small sample size, short follow-up period, and exclusive use of patient reports to measure the indicators of treatment engagement. In addition, it is not clear which component of the intervention—the patient-centered assessment process with patients, or the feedback to staff and discussion of how to modify treatment plans based on a patient’s personality profile—contributed to the higher ratings of program adjustment at the 1-month mark. Although testing of this intervention is still in the preliminary stages, if evidence accumulates to support its efficacy, research designs should evaluate the incremental contribution of the staff feedback component of the intervention to determine its association with outcomes, above and beyond the impact of providing feedback to patients only.

In terms of treatment outcomes, although the impacts of the intervention on length of stay and premature dropout were in the expected directions, they were not statistically significant. Notwithstanding the fact that power was limited in this small sample, it is possible that the dosing of the intervention may need to be increased in order to have a more beneficial impact on treatment outcomes. For example, an additional follow-up session and/or reassessment of patients’ personality at the end of treatment may boost the positive 1-month effects and provide patients with data on progress toward their treatment goals. Notably, this modification would align the intervention with measurement-based models of care, which have shown promise in the treatment of SUD patients [27]. Further, future work should examine the impact of the intervention on treatment outcomes beyond 1 month, particularly post-treatment outcomes such as engagement in continuing care, substance use, and functioning.

## Conclusions

The findings of this pilot RCT highlight the clinical utility of providing SUD patients with patient-centered feedback based on the results of personality testing, and provide preliminary support for the acceptability and efficacy of this intervention to facilitate early engagement in residential treatment. Therefore, this work supports future studies (e.g., a larger Stage II trial) [21] aimed at testing the efficacy of this brief, patient-centered intervention to increase retention in SUD care, as well as improve post-treatment outcomes related to substance use and general mental health functioning.

## Appendix

### Your Personality Summary

Your statements on the questionnaire you completed were compared with others your age and gender to show your particular ways of thinking, feeling, and interacting with others. The results that may be most interesting to you, given the question(s) you had, as well as how you can best adjust to and benefit from your treatment program, are listed below:

**Your assessment question:** “How do I compare to others in terms of self-esteem and anger?*”*

1. **Your feelings of self-worth and competence are similar to those of others your age; however, you are more likely than others to believe that you cannot cope with stress and will fall apart under pressure.**
  Possible adjustment to the program:
  - May often feel overwhelmed by treatment program assignments and duties.
  - May doubt self and capabilities when facing challenging situations.
  Recommendations:
  - Practice relaxation techniques when feeling stressed; ask for support from peers and staff if feeling overwhelmed with your duties.
  - Complete cognitive restructuring worksheets (“Triple Columns”) on statements that reflect your beliefs in your ability to cope with stress (e.g., “If I left the program, I would die.”)
2. **You tend to be more assertive, outspoken, and prone to conflict than others. You tend to struggle with “letting things go” when bothered by the actions of others.**
  Possible adjustment to the program:
  - Likely to adjust well to program expectation of providing feedback to peers.
  - May easily get involved in interpersonal conflict and drama with other residents.
  Recommendations:
  - Log when you have insisted on your way, and when you followed another's way.
  - List past instances of involvement in conflict with others, how you acted, and the pros & cons of your behavior.
  - Attend interpersonal skills groups (e.g., Dialectical Behavior Therapy [DBT]).
3. **Compared to your peers, you have more difficulty trusting others.**
  Possible adjustment to the program:
  - May have difficulty confiding in staff or peers.
  - May struggle to form relationships with others or accept their feedback or advice.
  - May distrust others and believe they have harmful intentions, which could lead to altercations.
  Recommendations:
  - List pros and cons of trusting others. Log instances when you have and have not benefitted from trusting someone else’s advice.
  - “Triple columns” on statements that reflect your beliefs in the trustworthiness of others.
4. **You are less likely than others to plan ahead and tend to act impulsively, particularly when you feel stressed out.**
  Possible adjustment to the program:
  - May be stressed by the high structure and planning requirements of the program.
  - May receive critical feedback for not “thinking before acting.”
  Recommendations:
  - Consider past spur-of-the-moment decisions and the pros and cons of them.
  - Log impulsive thoughts & feelings; use “Triple Columns” to manage impulses and develop alternative responses.
